# Comparison of Cricothyroid Membrane Puncture Anesthesia and Topical Anesthesia for Awake Fiberoptic Intubation: A Double-Blinded Randomized Controlled Trial

**DOI:** 10.3389/fmed.2021.743009

**Published:** 2021-11-16

**Authors:** Shaocheng Wang, Chaoli Hu, Tingting Zhang, Xuan Zhao, Cheng Li

**Affiliations:** ^1^Department of Anesthesiology, Shanghai Pulmonary Hospital, School of Medicine, Tongji University, Shanghai, China; ^2^Department of Anesthesiology and Perioperative Medicine, Shanghai Fourth People's Hospital, School of Medicine, Tongji University, Shanghai, China; ^3^Department of Anesthesiology, Shanghai Tenth People's Hospital, School of Medicine, Tongji University, Shanghai, China; ^4^Translational Research Institute of Brain and Brain-Like Intelligence, Shanghai Fourth People's Hospital, School of Medicine, Tongji University, Shanghai, China; ^5^Clinical Research Center for Anesthesiology and Perioperative Medicine, Tongji University, Shanghai, China

**Keywords:** cricothyroid membrane puncture anesthesia, topical anesthesia, difficult airway, awake fiberoptic intubation (AFOI), dexmedetomidine (DEX), sufentanil

## Abstract

**Background:** Awake fiberoptic intubation (AFOI) is commonly used for patients with a difficult airway. The purpose of this study was to evaluate the efficacy of cricothyroid membrane puncture anesthesia and topical anesthesia during AFOI.

**Methods:** A total of 70 patients (the American Society of Anesthesiologists score I-III) with anticipated difficult airways scheduled for nonemergency surgery with AFOI were randomly slated to receive cricothyroid membrane puncture anesthesia (*n* = 35) or topical anesthesia (*n* = 35). Each group received dexmedetomidine at a dose of 1.0 μg/kg and sufentanil at a dose of 0.2 μg/kg over 10 min for conscious sedation before intubation. The endoscopy intubation, post-intubation condition, and endoscopy tolerance as scored by the anesthetists were observed. The satisfaction of the operator regarding the procedure and the satisfaction of the patient 24 h after the surgery were also recorded. We recorded the success rate of the first intubation, intubation time, and hemodynamic changes during the procedure and also the adverse events.

**Results:** Better intubation scores, operator satisfaction, and satisfaction of the patient were observed in the cricothyroid membrane puncture anesthesia group than in the topical anesthesia group (*p* < 0.05). The intubation time in the cricothyroid membrane puncture anesthesia group was less than that in the topical anesthesia group (*p* < 0.05). There were no significant differences in the patient tolerance scores, the success rate of the first intubation, hemodynamic changes, and adverse events between both the groups.

**Conclusion:** Compared with topical anesthesia, cricothyroid membrane puncture anesthesia provided better intubation conditions and less intubation time with greater satisfaction of the patient and operator during endoscopic intubation.

**Clinical Trial Registration:** URL: http://www.chictr.org.cn/showproj.aspx?proj=42636, Identifier: ChiCTR 1900025820.

## Introduction

The incidence of the difficult airway during clinical anesthesia is as high as 4.5–7.5% (1); this is a significant issue, as failure to maintain an unobstructed patient airway may lead to hypoxemia, brain damage, or even death (2). Awake fiberoptic intubation (AFOI) is an effective technique for the patients with difficult airways; it is considered the gold standard among intubation techniques (3, 4). Optimal intubation conditions for AFOI are as follows: the patient should be comfortable, cooperative, and have hemodynamic stability; moreover, the anesthesiologist must be able to maintain the airway of the patient with spontaneous ventilation (5). To achieve these conditions, adequate conscious sedation and high-grade local anesthesia are required. Previous studies have demonstrated that sufentanil and dexmedetomidine provide effective sedation during AFOI without depressing respiratory function (6–11).

The two most commonly used local anesthesia techniques are: cricothyroid membrane puncture anesthesia and topical anesthesia that can provide reasonable levels of safety and comfort (12–15). The duration of AFOI should be kept as short as possible to minimize the patient discomfort. Compared with the topical anesthesia performed by using the spray-as-you-go technique (16, 17), cricothyroid membrane puncture anesthesia seems to be faster and more effective (18–21). To the best of our knowledge, there is no previous study that has compared topical anesthesia and cricothyroid membrane puncture anesthesia in awake fiberoptic nasotracheal intubation. Therefore, this study was designed to compare the efficacy of the topical anesthesia and cricothyroid membrane puncture anesthesia in patients with difficult airway during AFOI.

## Materials and Methods

This study was approved by the Institutional Review Board of the Ethics Committee of Shanghai Tenth People's Hospital affiliated with the Shanghai Tongji University School of Medicine (SHSY-IEC-4.0/19-81-01). Written informed consent was obtained from each patient. It was registered as a clinical trial (www.chictr.org.cn, Identifier: ChiCTR 1900025820). We recruited 81 patients who were 18–80 years old and scheduled for AFOI due to an anticipated difficult airway with an American Society of Anesthesiologists (ASA) score of I-III. The exclusion criteria included the heart rate (HR) < 50 beats/min, systolic blood pressure (SBP) < 90 mm Hg, use of an α2-adrenoreceptor agonist or antagonist within the past 14 days, cirrhosis, nasal injury, nasal polyps, upper airway obstruction, skull base fracture, sinusitis, intracranial hypertension, heart failure, emergency surgery, coagulation disorders, contraindication to the performance of cricothyroid membrane puncture (thyroid swelling, local infection, or laryngeal disorder), allergic to related drugs and materials, cannot cooperate actively, and cannot objectively describe the symptoms. A total of 11 patients were excluded, seven patients met the exclusion criteria and four patients declined to participate. The remaining 70 patients were assigned (using a computer-generated randomization schedule) to receive topical anesthesia (Group A) or cricothyroid membrane puncture anesthesia (Group B). An anesthetist nurse generated the allocation sequence and assigned the patients to their groups, while another anesthetist nurse recorded the experimental and postoperative follow-up data. One anesthetist prepared the drug infusion, while another anesthetist was in charge of cricothyroid membrane puncture and intubation, graded intubation condition, and operator satisfaction. The patients, anesthetist nurses, and intubating anesthetist were all blinded to the group allocation.

Once the patient was transferred to the operation room, intravenous access was established and standard monitoring parameters (non-invasive blood pressure, pulse oximetry, and ECG) were recorded every 2 min. The patient inhaled oxygen through a nasal catheter (4 l/min). Topical anesthesia of the nasal cavity was initiated using 2 ml 2% lidocaine; simultaneously, 2 ml 1% ephedrine was instilled into the nasal cavity to contract the nasal vessels. Sufentanil (0.2 μg/kg) and dexmedetomidine (1 μg/kg) were diluted into 100 ml 0.9% saline and the patient received the drugs intravenously over 10 min. When drug infusion was completed, cricothyroid membrane puncture was performed by using a 23G needle. After verification of intratracheal placement by performing air aspiration, 3 ml of 2% lidocaine was injected in Group B, while 3 ml of 0.9% saline was injected in Group A. After injection, the patient was asked to cough to transport the local anesthetic from the tracheal injection site to the supraglottic mucosa. After a cricothyroid membrane puncture, a 30 sec wait was conducted as part of the protocol. When cricothyroid membrane puncture was accomplished, a fiberoptic scope (Olympus LF-DP 3.1 mm, Olympus, Tokyo, Japan) was loaded with a 7.0-mm tracheal tube for male patients or a 6.5-mm tube for female patients. Then, 2 ml of 2% lidocaine for Group A or 2 ml of 0.9% saline for Group B was sprayed directly onto the glottis through the channel of the fiberoptic scope once the glottic structures were identified. After a 1-min wait, another 2 ml of 2% lidocaine for Group A or 2 ml of 0.9% saline for Group B was sprayed below the vocal cords. After a further 1-min wait, the tracheal tube was slightly inserted *via* the fiberoptic scope tube. During intubation, if the peripheral oxygen saturation (SpO_2_) of the patient fell ≤ 92%, the procedure was halted and the patient was asked to take deep breaths. Another intubation attempt was made when the SpO_2_ was recovered to ≥ 95%. Hemodynamic changes (HR, mean arterial pressure, and pulse oximetry) were analyzed for both the groups at three time points (baseline, immediately after drug infusion, and immediately after intubation).

The primary outcomes included intubation times (from the begging of cricothyroid membrane puncture to the end of nasal tracheal intubation); intubation scores as assessed according to the vocal cord movement (1, open; 2, moving; 3, closing; 4, closed), coughing (1, none; 2, slight; 3, moderate; 4, severe), and limb movement (1, none; 2, slight; 3, moderate; 4, severe); patient tolerance as assessed using a five-point fiberoptic intubation comfort score (1, no reaction; 2, slight grimacing; 3, heavy grimacing; 4, verbal objection; 5, defensive movement of head or hands), and a three-point score assessed immediately after nasotracheal intubation (1, cooperative; 2, minimal resistance; 3, severe resistance); and first intubation attempt success rate.

Other parameters during intubation included the satisfaction of the operator regarding the intubation process (0, completely dissatisfied; 10, completely satisfied) and the occurrence of a hypoxic episode (SpO_2_ < 92%). All the adverse events were recorded.

## Statistical Analysis

We used the GraphPad Prism version 8.0 (GraphPad Software Inc., San Diego, California, USA) for the statistical analyses. Continuous variables are described as mean ± SD and were compared by using the paired *t*-test. The chi-squared test or the Fisher's exact test was used to compare categorical variables between the groups. Intubation conditions and tolerance score were analyzed using the independent samples Mann–Whitney *U* test. Blood pressure and HR at different time points were compared by using the two-way repeated measures analysis of variance. A *p* < 0.05 was regarded as statistically significant.

## Results

**Figure 1:**
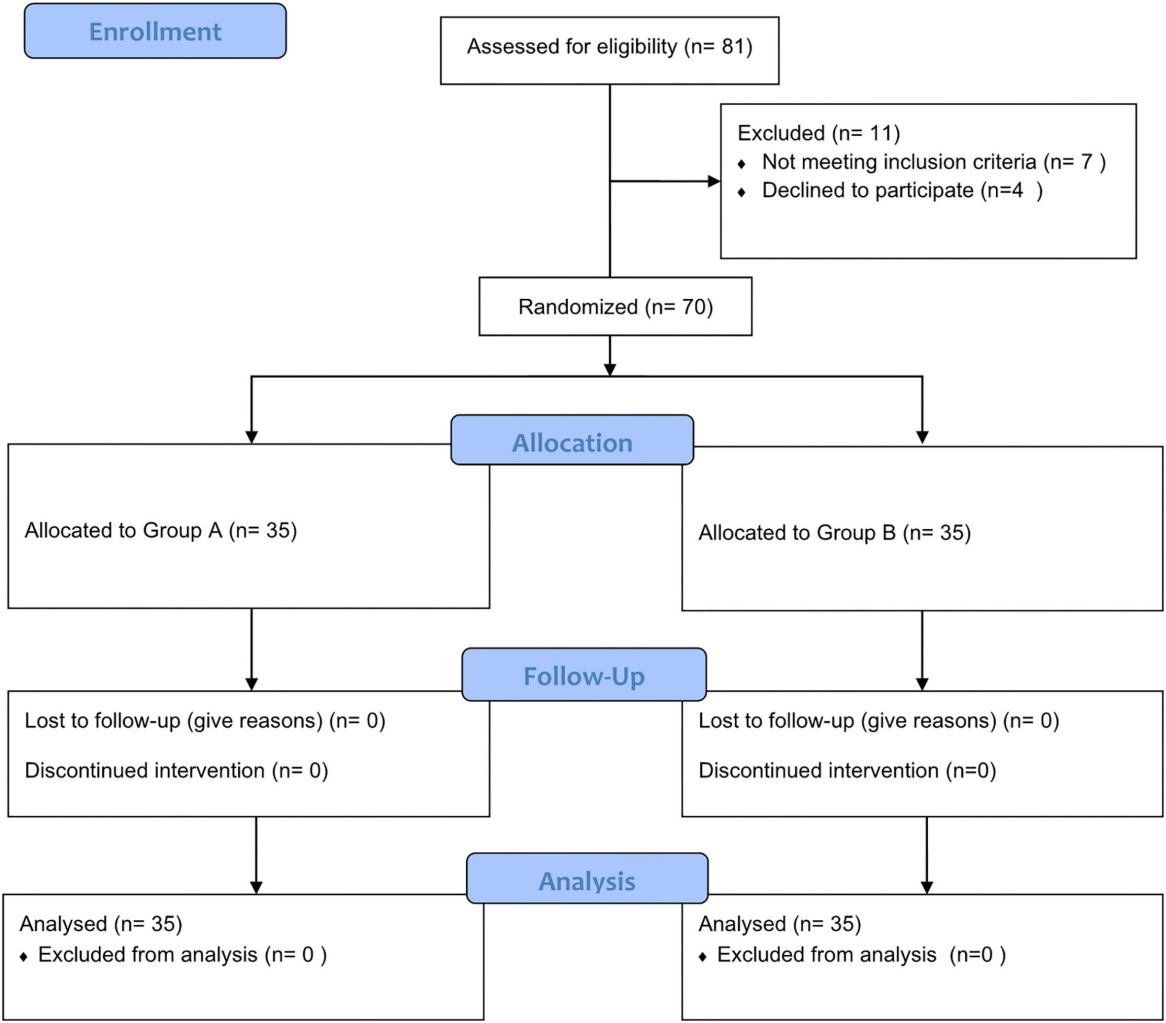
Flowchart for the study recruitment.

A total of 70 patients (33 males and 37 females) with anticipated difficult airways were enrolled in this study (Figure 1). The baseline data of the two groups showed no differences (Table 1).

**Table 1:** Demographic and clinical characteristics of study participants.

**Characteristic**	**Group A** **(*n* = 35)**	**Group B** **(*n* = 35)**	***P* value**
Age (years)	57.6 ± 9.8	56.7 ± 11.9	0.734
Sex (male/female)	16/19	17/18	0.811
Weight (Kg)	66.2 ± 9.8	65.2 ± 10.1	0.657
ASA status (1/2/3)	16/16/3	12/20/3	0.602
Mallampatti grade (2/3/4)	6/19/10	8/20/7	0.841
Mouth opening (cm)	3.6 ± 0.6	3.8 ± 0.7	0.184
RSS score (1/2)	8/27	6/29	0.766

*Data are presented as mean ± standard (SD) or number*.

*ASA, American Society of Anesthesiologists*.

The Ramsay Sedation Scale (RSS) score after the drug infusion showed no significant differences between both the groups. All the patients were successfully intubated with AFOI. There was no significant difference between both groups in the first intubation attempt success rate, but the first intubation attempt failed in two patients from Group A. The intubation time in Group B (200.4 ± 28.1) was lower than in Group A (244.1 ± 91.5) (*p* = 0.003). The intubation scores were better in Group B compared with Group A with regard to the vocal cord movement, cough, limb movement, and operator satisfaction (*p* < 0.05), but there was no significant difference in the patient tolerance score and comfort score of the patients after intubation (Table 2).

**Table 2:** Airway management characteristics.

	**Group A** **(*n* = 35)**	**Group B** **(*n* = 35)**	***P* value**
RSS after drug infusion (2/3)	9/26	12/23	0.603
First intubation attempt success rate (%)	94.286	100	0.151
Intubation time (sec)	244.1 ± 91.5	200.4 ± 28.1	0.003
**Intubation scores**			
Vocal cord movement 1/2/3/4	12/17/4/0	22/12/1/0	0.01
Cough 1/2/3/4	14/18/3/0	27/8/0/0	0.002
Limb movement 1/2/3/4	22/6/7/0	29/5/1/0	0.002
Patient tolerance 1/2/3/4	10/20/5/0	17/17/1/0	0.29
Patients' comfort score after intubation 1/2/3/4	24/10/1/0	30/4/1/0	0.267
Operator's satisfaction (1–10)	7 ± 0.5	8.9 ± 0.9	0.0001

*Data are presented as mean ± standard (SD) or number*.

*RSS, Ramsay sedation score*.

**Figure 2:**
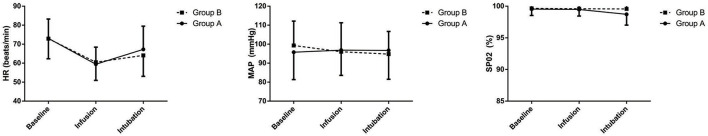
Heart rate (HR), mean arterial blood pressure (MAP), and peripheral oxygen saturation (SpO_2_) before premedication (baseline), immediately after drug infusion (infusion), and immediately after intubation (intubation). Group A: Topical anesthesia; Group B: Cricothyroid membrane puncture anesthesia. Points are expressed as mean ± SD.

There was no significant difference in the hemodynamic change between both the groups at three time points: baseline, immediately after drug infusion, and immediately after intubation (Figure 2).

The incidence of adverse events (hypertension, hypotension, tachycardia, bradycardia, and hypoxia) was not significantly different between both the groups. Postanesthetic interview parameters: hoarseness, sore throat, and recall of intubation did not differ between both the groups, but patient satisfaction in Group B (9.4 ± 0.8) was better compared with that in Group A (7.5 ± 1.3) (*p* = 0.0007) (Table 3).

**Table 3:** Postoperative visit data and advent events.

	**Group A** **(*n* = 35)**	**Group B** **(*n* = 35)**	***P* value**
Hoarseness	1	1	1
Sore throat	1	3	0.614
Recall of intubation	25	30	0.244
Patients' satisfaction	7.5 ± 1.3	9.4 ± 0.8	0.0007
Hypertension	0	0	1
hypotension	0	0	1
tachycardia	1	1	1
bradycardia	2	3	0.69
hypoxia	0	0	1

*Data are presented as mean ± standard (SD) or number*.

## Discussion

This study aimed to compare the two techniques of topical anesthesia that are both deemed effective during AFOI in patients with anticipated difficult airways. To the best of our knowledge, our study is the first to compare cricothyroid membrane puncture anesthesia and topical anesthesia in awake fiberoptic nasotracheal intubation.

In the existing literature on AFOI, several viewpoints exist. Some studies mention that cricothyroid membrane puncture anesthesia is a more effective method than topical anesthesia using the spray-as-you-go technique (18, 22), while others state that the spray-as-you-go technique is superior to the cricothyroid membrane puncture technique (23).

This study showed that both the cricothyroid membrane puncture anesthesia and topical anesthesia were effective for AFOI, but the cricothyroid membrane puncture anesthesia technique required less intubation time and led to better intubation scores, a higher success rate of first intubation, and a higher satisfaction score for both operator and patients during AFOI.

We chose the intubation time as the primary outcome of our study, because it is the clearest criterion to evaluate the efficiency of AFOI. Along with an adequate analgesia and sedation, we believe that a rapid AFOI procedure is important for ensuring safety and comfort of the patient. Our study found that the cricothyroid membrane puncture anesthesia technique was significantly faster than the topical anesthesia technique; this is consistent with the results of a previous study (24). Moreover, to ensure the double blindness of the experiment, the cricothyroid membrane puncture anesthesia group also used the spray-as-you-go technique for comparison with topical anesthesia; this increased the intubation time by 2 min and might have reduced patient comfort and satisfaction.

The secondary outcomes were the intubation scores during AFOI. Scoring systems similar to the one we used to evaluate intubation scores are described in the literature (6, 25, 26), but the results were different in those studies. Our study showed that the cricothyroid membrane puncture anesthesia technique was better than the topical anesthesia technique as it exhibited better intubation scores, higher success rate of first intubation, and higher operator satisfaction score. Although cricothyroid membrane puncture anesthesia was administered subglottically, it provided a better topical block. Despite the fact that the patient tolerance and patient comfort scores were better in the cricothyroid membrane puncture anesthesia group, this difference did not achieve statistical significance.

We knew from our experience and from the literature (27–29) that adequate analgesia and sedation are necessary for AFOI. Therefore, we administered sufentanil (0.2 μg/kg) and dexmedetomidine (1 μg/kg) over 10 min before AFOI to obtain sufficient analgesia and sedation. On following this procedure, all the patients had an RSS score > 1 after the drug infusion and no patient felt obvious pain in any of the AFOI procedures; this result is consistent with those of the previous studies (30–32).

Hemodynamic stability is a measure of stress response during AFOI. We did not find any significant difference between the two groups regarding this factor. This finding is consistent with the results of the previous studies, wherein AFOI performed by experienced operators had no influence on the hemodynamic stability of the patient (33, 34).

Dexmedetomidine has been reported to decrease noradrenaline release and centrally mediated sympathetic tone (35). However, it may cause side effects including hypotension, bradycardia, and hypoxia (36). In our study, five patients developed bradycardia after drug infusion that can be treated easily with atropine and two patients developed tachycardia after intubation. There was no significant difference between the two groups regarding the adverse effects of dexmedetomidine.

Postoperative visit data showed the majority of the patients had no hoarseness or soreness of the throat. The cricothyroid membrane puncture anesthesia group exhibited higher patient satisfaction and less recall of intubation than the topical anesthesia group. Therefore, we conclude that cricothyroid membrane puncture anesthesia technique, if performed by experienced anesthesiologists, provide a high level of comfort to the patient.

This study had some limitations. There were only 70 patients enrolled in our study, further larger sample studies are required to confirm our results. Another limitation was the lack of dose-effect study; the doses of sufentanil and dexmedetomidine used were based on data provided in the previous studies. Our drug infusion protocol is not suitable for the emergency operation, as it requires more than 10 min. Despite sufficient analgesia and sedation, cricothyroid membrane puncture is still an invasive operation.

## Conclusion

Both the techniques of topical anesthesia are effective in AFOI, but cricothyroid membrane puncture anesthesia provided better intubation conditions and less intubation time with greater satisfaction of the patient and operator when compared with topical anesthesia.

## Data Availability Statement

The original contributions presented in the study are included in the article/supplementary material, further inquiries can be directed to the corresponding author/s.

## Ethics Statement

The studies involving human participants were reviewed and approved by Review Board of the Ethics Committee of Shanghai Tenth People's Hospital affiliated with Shanghai Tongji University School of Medicine (SHSY-IEC-4.0/19-81-01). The patients/participants provided their written informed consent to participate in this study.

## Author Contributions

SW, CH, TZ, XZ, and CL: provision of study materials or patients, collection and assembly of data, and final approval of manuscript. CL: administrative support. SW, CH, XZ, and CL: conception and design, data analysis and interpretation, and manuscript writing. All authors contributed to the article and approved the submitted version.

## Funding

This study was supported by the National Natural Science Foundation of China (Grant Number 81600921) located in No. 83 Shuangqing Road, Haidian District, Beijing, 100085, China and the Natural Science Foundation of Shanghai (Grant Number 20ZR1442900) located in No. 200, Renmin Avenue, Shanghai, 200003, China.

## Conflict of Interest

The authors declare that the research was conducted in the absence of any commercial or financial relationships that could be construed as a potential conflict of interest.

## Publisher's Note

All claims expressed in this article are solely those of the authors and do not necessarily represent those of their affiliated organizations, or those of the publisher, the editors and the reviewers. Any product that may be evaluated in this article, or claim that may be made by its manufacturer, is not guaranteed or endorsed by the publisher.
